# Supplementary material for: Calcium-Free and Cytochalasin B Treatment Inhibits Blastomere Fusion in 2-Cell Stage Embryos for the Generation of Floxed Mice via Sequential Electroporation
Source: Cells. 2020 Apr 28;9(5):1088. doi: 10.3390/cells9051088 (PMC7290713; doi:10.3390/cells9051088)
Supplement: Supplementary file 1 [file cells-09-01088-s001.pdf]

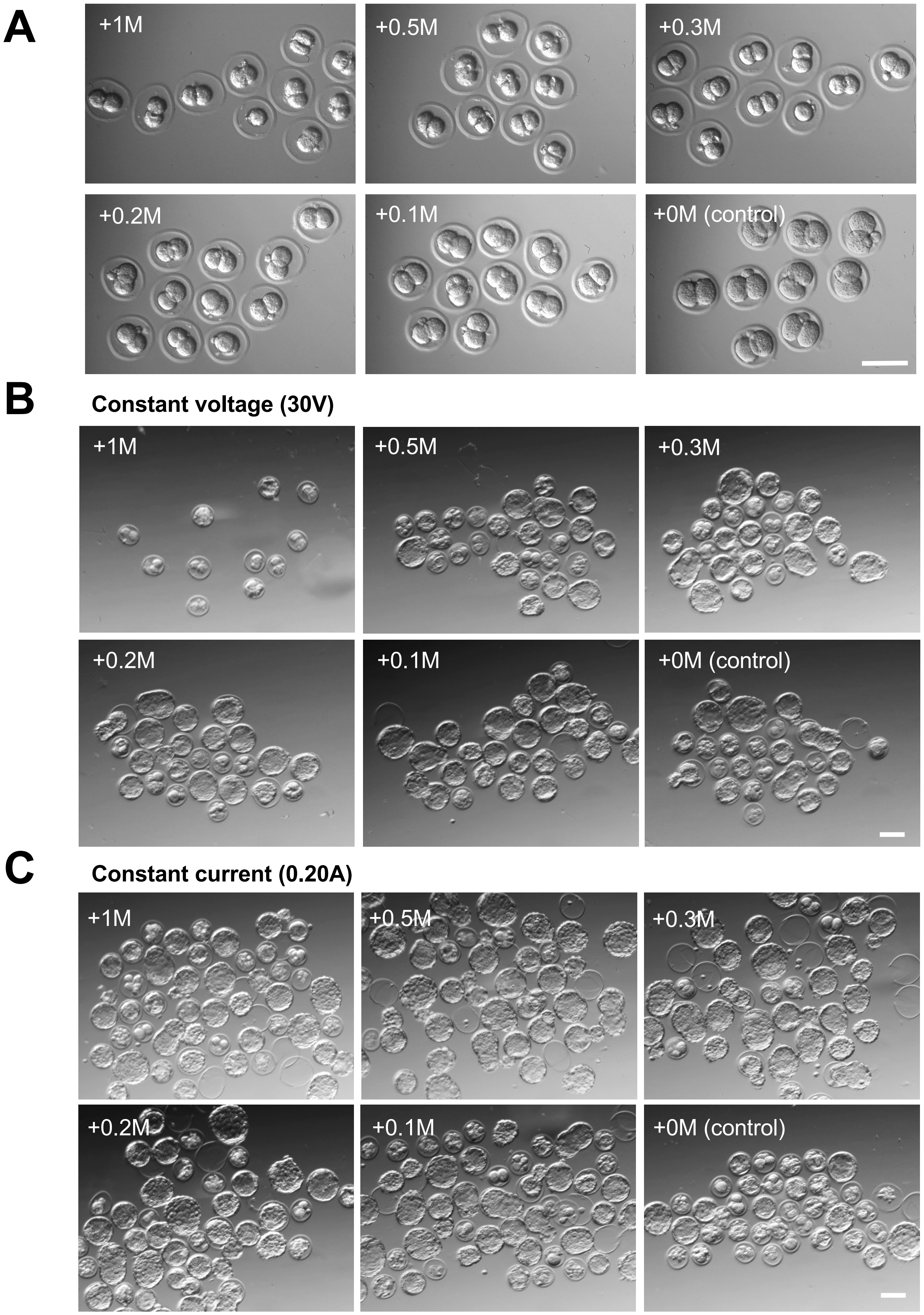

Figure S1. The morphology of embryos by hypertonic treatment. (**A**) 2-cell stage embryos after treatment with several hypertonic conditions. (**B**) *In vitro* development of embryos after 2-cell EP under constant voltage (30V). (**C**) Those under constant current (0.20A). Scale bars, 100 μm.

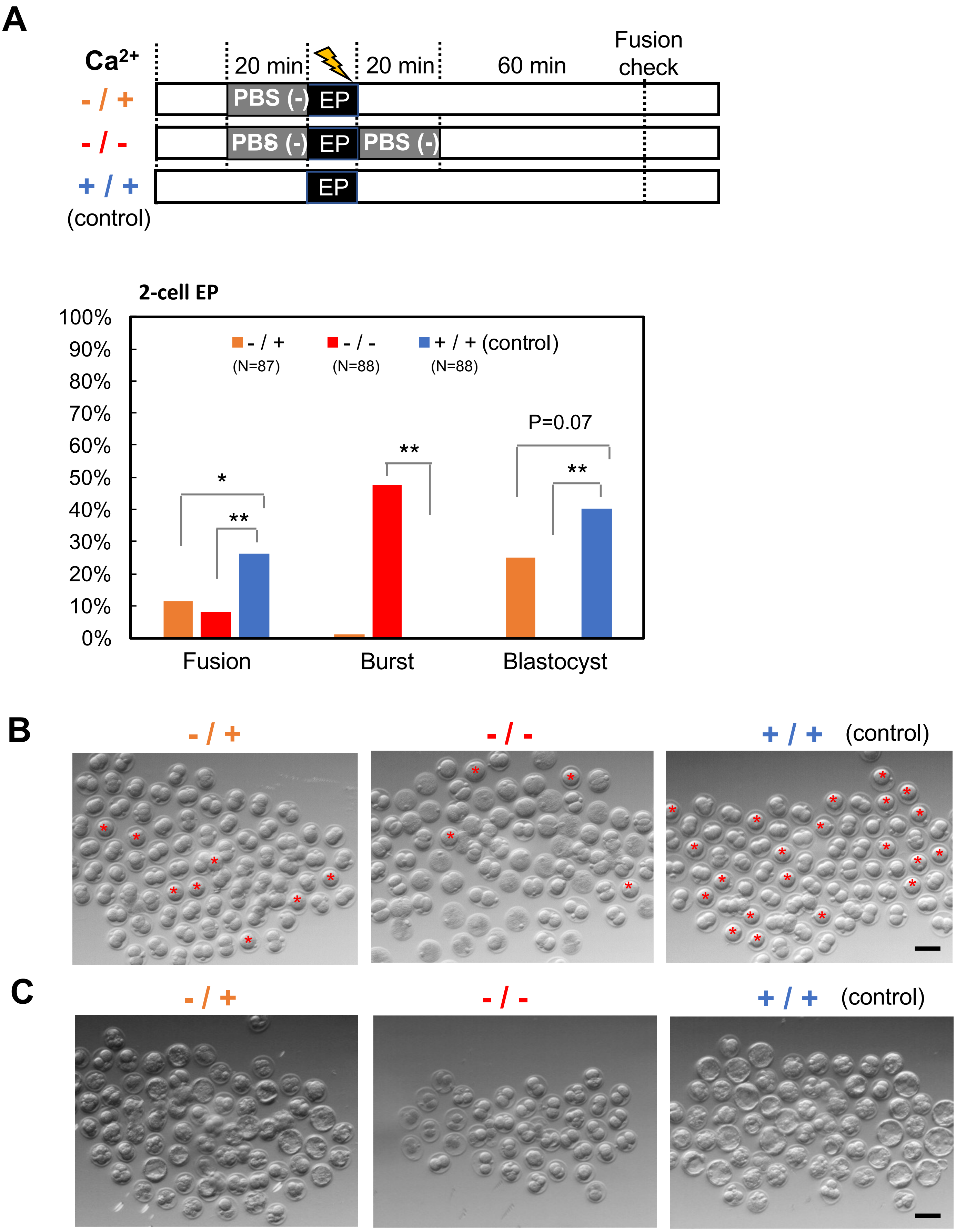

Figure S2. Effects of PBS(-) treatment on blastomere fusion and embryonic development of 2-cell EP. (**A**) Fusion, burst, and development (blastocyst) rate by 2-cell EP. (**B**) Morphology of 2-cell EP embryos. Asterisks indicate fused embryos. (**C**) Morphology of 2-cell embryos in 3 days of culture post-EP. N indicates the number of 2-cell stage embryos used for the analysis. *P < 0.05, **P < 0.01 (compared with control). Scale bars, 100 μm.

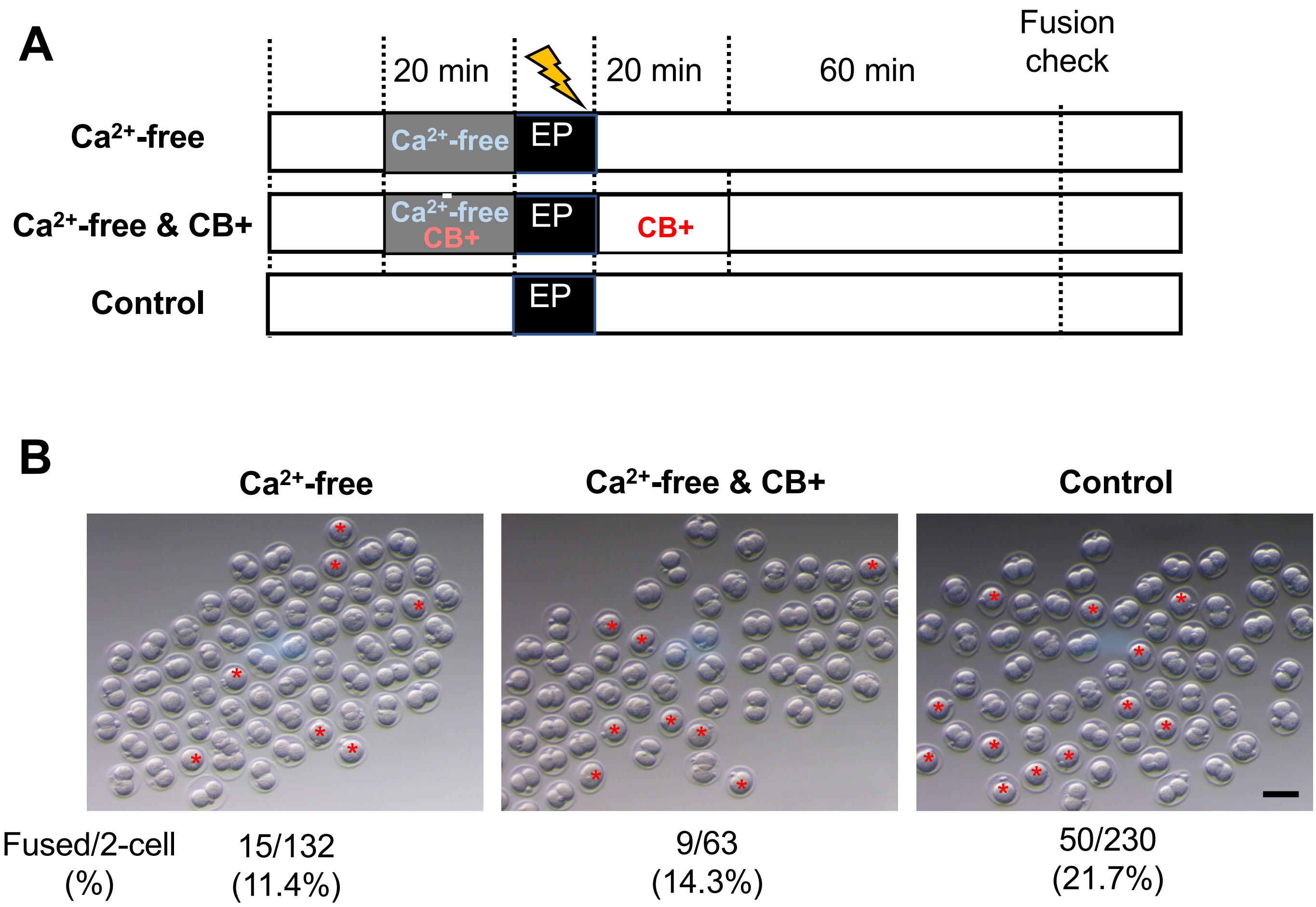

Figure S3 The combined Ca^{2+}-free and CB treatment does not have a better effect on blastomere fusion than the only Ca^{2+}-free treatment. (**A**) Experimental timeline of Ca^{2+}-free and CB treatment. (**B**) Morphology of 2-cell EP embryos. Asterisks indicate fused embryos. Scale bars, 100 μm.
